# Using multi-level regression to determine associations and estimate causes and effects in clinical anesthesia due to patient, practitioner and hospital or health system practice variability

**DOI:** 10.1007/s00540-024-03408-3

**Published:** 2024-09-18

**Authors:** Kazuyoshi Aoyama, Alan Yang, Ruxandra Pinto, Joel G. Ray, Andrea Hill, Damon C. Scales, Robert A. Fowler

**Affiliations:** 1 Department of Anesthesia and Pain Medicine, The Hospital for Sick Children, 555 University Ave, #2211, Toronto, ON M5G 1X8 Canada; 2 Program in Child Health Evaluative Sciences, SickKids Research Institute, Toronto, Canada; 3 Department of Critical Care Medicine, Sunnybrook Health Science Center, Toronto, Canada; 4 Sunnybrook Research Institute, Sunnybrook Health Science Center, Toronto, Canada; 5 Keenan Research Centre of the Li Ka Shing Knowledge Institute of St. Michael’s Hospital, Toronto, Canada; 6 Department of Obstetrics and Gynecology, St. Michael’s Hospital, Toronto, Canada; 7 Institute of Health Policy, Evaluation and Management, University of Toronto, Toronto, Canada

**Keywords:** Big data, Clinical anesthesia research, Clustering, Multi-level regression models, Variability

## Abstract

In this research methods tutorial of clinical anesthesia, we will explore techniques to estimate the influence of a myriad of factors on patient outcomes. Big data that contain information on *patients*, treated by *individual anesthesiologists and surgical teams*, at different *hospitals*, have an inherent multi-level data structure (Fig. 1). While researchers often attempt to determine the association between patient factors and outcomes, that does not provide clinicians with the whole story. Patient care is *clustered* together according to clinicians and hospitals where they receive treatment. Therefore, multi-level regression models are needed to validly estimate the influence of each factor at each level. In addition, we will explore how to estimate the influence that *variability*—for example, one anesthesiologist deciding to do one thing, while another takes a different approach—has on outcomes for patients, using the *intra-class correlation coefficient* for continuous outcomes and the *median odds ratio* for binary outcomes. From this tutorial, you should acquire a clearer understanding of how to perform and interpret multi-level regression modeling and estimate the influence of variable clinical practices on patient outcomes in order to answer common but complex clinical questions.

**Fig. 1:**
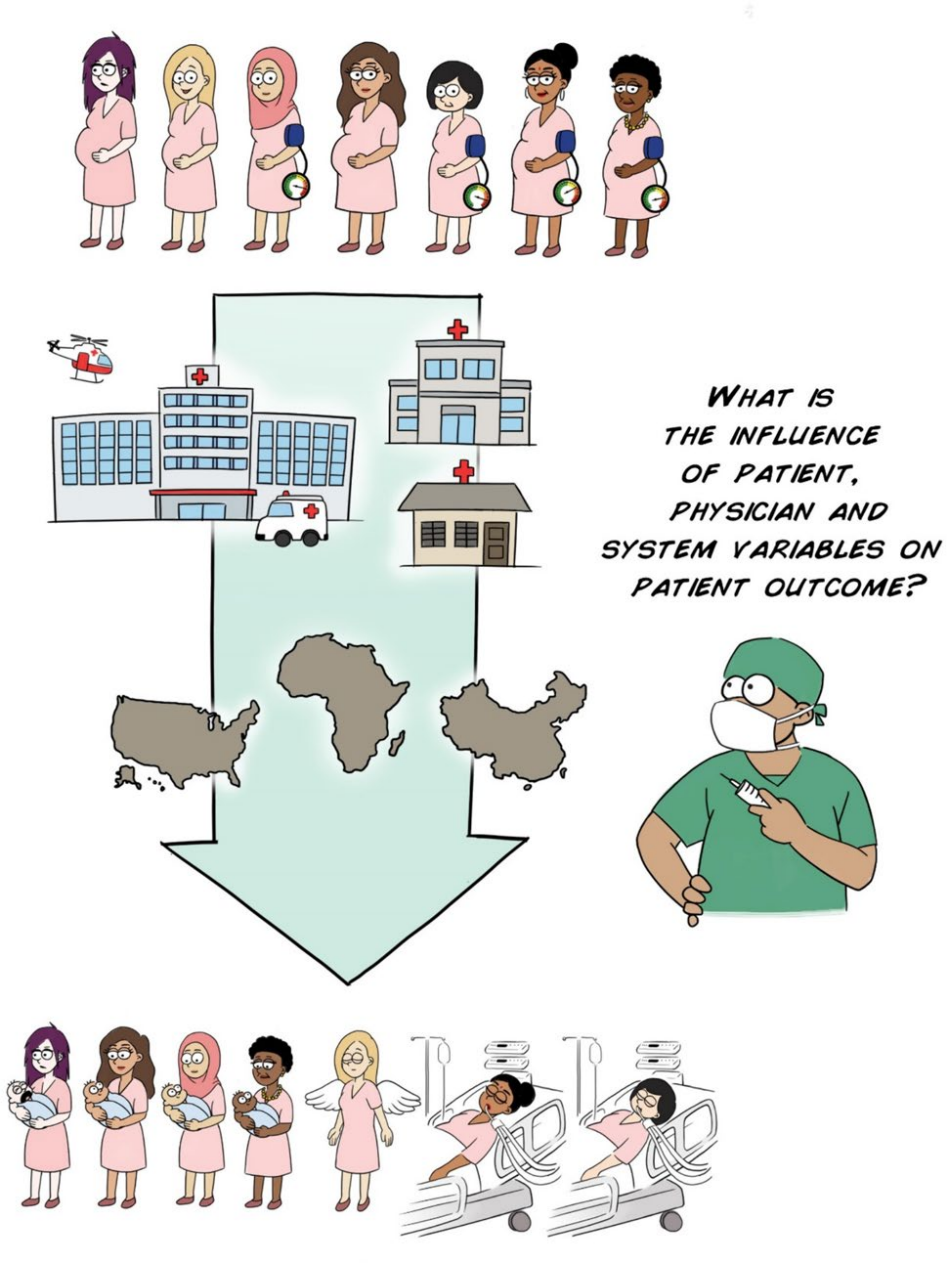
Infographics

Infographics

## Clinical scenario

**Fig. 2:**
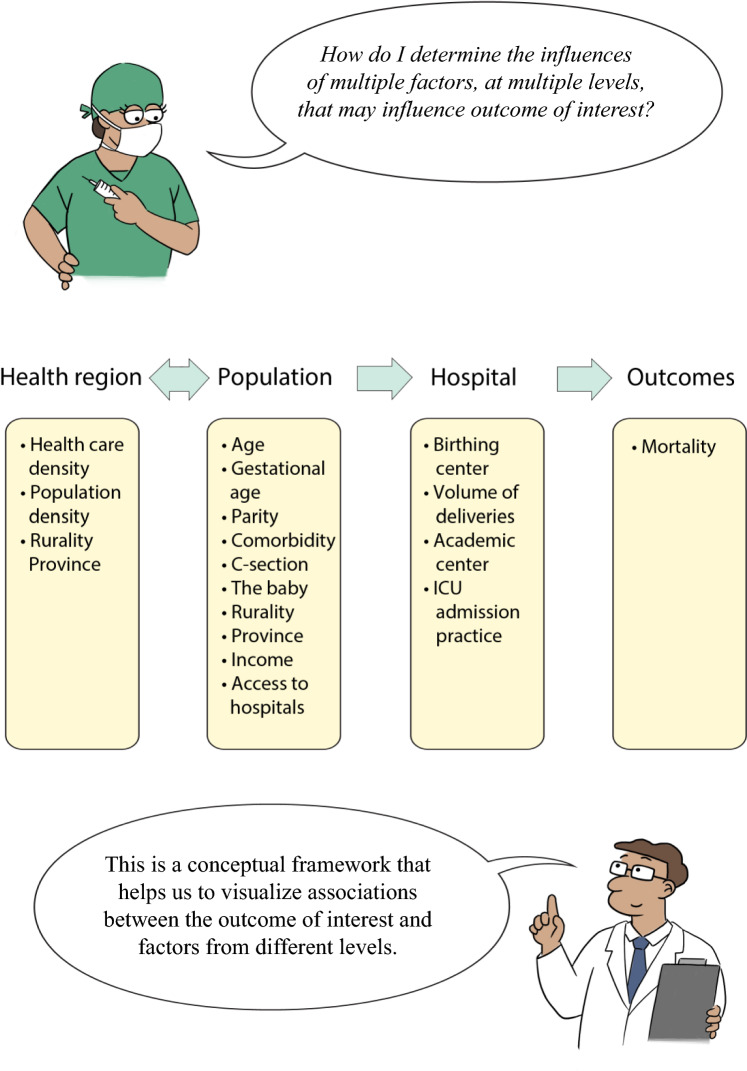
Conceptual framework of multi-level factors

We previously published another tutorial, which described how to determine associations with regression models [1]. Therefore, we presented the unfortunate case of a woman with peri-partum multi-organ failure who was transferred from an outlying hospital to an emergency room and intensive care unit (ICU). While this woman had a history of obesity, hypertension, and gestational diabetes mellitus, we wondered whether there were other non-patient factors that influenced her outcome, for example, the care she received during her pregnancy, how far she lived from her referring hospital, and whether her referring hospital had an ICU and a health care team experienced in managing high-risk pregnancies. How can we estimate the influence of these factors at different levels, specifically, at the level of the (1) patient, (2) provider(s), (3) hospital and (4) health region (Fig. 2)? What is the influence of variability in clinical practice—say criteria for admission to the ICU—on patient outcomes? To address this question with more than speculation, we typically need data on a lot of patients, including information on the care they received, care providers, and the healthcare settings involved. This introduces the relevance of evaluating patient outcomes and studying different potential predictors in a multi-level setting.

## Determining associations with regression models

We begin with estimating the influence of patient factors on clinical outcome only at the patient level. During this procedure, *bias*, which is any systematic error occurring in the design or conduct of clinical research that should be accounted for. We should also account for *confounding*, which is when variables are related to the predictors yet have their own association with the outcome [2]. While one approach to mitigate confounding is to design a randomized clinical trial [3], another approach, using observational studies, is regression [1, 2]. When the outcome is continuous, a *linear* regression equation establishes a relationship between the outcome variable and the exposure variables, or predictors. *Logistic* regression is used when the outcome is binary. The association of multiple exposure variables with the outcome variable can be assessed simultaneously, by assigning relative magnitudes of associations in a “multivariable” regression model. Here, we employ regression to evaluate the *association* between an exposure and an outcome of interest, accounting for potential confounding.

## Multi-level modeling

**Fig. 3:**
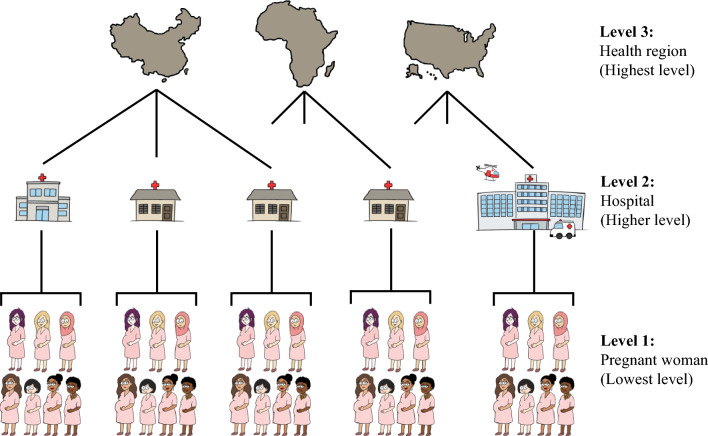
Diagram of hierarchical structure

*Multi-level* or *hierarchical* regression has different names: hierarchical linear regression models, random coefficient models, or mixed-models [4]. Herein, we use the term ‘multi-level regression’, which assumes a hierarchical structure to the data, with the outcome measured at the ‘lowest’ level of the hierarchy (usually patient level), and predictors measured at different levels (Fig. 3) [4–6].

When should we consider multi-level regression? A key consideration is the need to search for an association across a range of factors at different levels, and all of which may have some relation to the unit of analysis—typically an individual patient, though it could also be a hospital, clinic, provider specialty, or region [4–10]. We have two major approaches to using multi-level regression models: generalized estimating equation (GEE) and mixed-modeling [4]. Both are employed to adjust for hierarchical effects, or clustering effects. GEEs are known as population average models because they estimate a non-varying (i.e., average) coefficient among clusters. Although GEE can model variables up to two levels, mixed models can incorporate more than two levels. Mixed models estimate aspects of the model that vary by cluster; it can quantify variability among clusters and explore associations of such variability with the outcome at different levels, for example (Table 1). A detailed explanation of each approach is in literature [6]. Since one aspect of our research question seeks to quantify variability among clusters, we will employ mixed models for our multi-level regression and focus on mixed models in this paper.

**Table 1:** Key differences between generalized estimating equations and mixed models

Generalized estimating equations	Mixed models
Estimate an average coefficient among clusters	Estimate aspects of the model that vary by group
Accommodates variables at up to two levels	Accommodates variables at more than two levels
Specifies a covariance structure to account for within-cluster correlation	Quantifies variability among clusters
Distributional assumption of observed data and random effects	Absence of assumptions for how the coefficients vary

We first introduce a basic two-level regression model to better understand the concept. For a binary outcome like occurrence of preeclampsia (occurs or not), we use a *multivariable logistic* regression model to determine the relative contribution of different variables at different levels. Suppose we have a dataset from *j* hospitals, with a different number of pregnant patients, *n*_*j*_, in each hospital. The outcome is preeclampsia, measured at the patient (‘lowest’) level. In addition to considering the only patient-level predictor (presence of obesity), we wondered if hospitals having a dedicated obesity-in-pregnancy and birth center (modeled as a hospital-level predictor) would influence the risk of preeclampsia. To analyze these data, we could theoretically establish separate equations for each separate hospital to predict preeclampsia using obesity. We could next see how consistent the measures of association are across these hospital-specific equations to get a qualitative sense of whether differences may exist among hospitals. However, if we want to advance this further, and gain a quantitative estimate of the association of ‘hospital’ on the risk of preeclampsia, we can create the following model:

$$ \log \left( {\frac{{{\text{P}}\left( { {\text{Preeclampsia}} _{ij} } \right)}}{{1 - {\text{P}}\left( { {\text{Preeclampsia}} _{ij} } \right)}}} \right) = \, \beta_{0j} \, + \,\beta_{1} *{\text{obesity}}_{ij} $$

In a fixed-effect logistic regression model, the β’s are fixed parameters with β_0_ representing the model intercept (the log-odds of preeclampsia when obesity is not present) and β_1_ representing the model slope which quantifies the effect of obesity on the outcome. In the above model, the additional subscript *j* represents different hospitals (*j* = 1 …… *J*) and the subscript *i* represents individual patients (*i* = 1……*n*_*j*_). What differs from the fixed-effect model is that each hospital has a different intercept, β_0*j*_, but shares a common fixed slope, β_1_. We assume the intercept varies randomly across hospitals, hence they are random variables and are called “random intercepts”. These random intercepts are hospital level, which implies individuals within the same hospital share the same intercept, but intercepts are different for individuals at different hospitals. The next step might be to clarify the degree of variation of the random intercepts, β_*0j*_, at the hospital level:

$$ \beta_{0j} \, = \,\gamma_{00} \, + \,u_{0j} $$

This equation predicts the likelihood of preeclampsia, without obesity, in a hospital with an obesity-in-pregnancy and birth center (specifically, the ${j}^{th}$ hospital) using a common intercept γ_*00*_ and a hospital-specific error term u_*0j*_. The error term is normally distributed with mean being 0 and variance being σ^2^.

Then, our multi-level model can be expressed with a slightly more complicated, but more appropriate equation:

$$ \log \left( {\frac{{{\text{P}}\left( { {\text{Preeclampsia}} _{ij} } \right)}}{{1 - {\text{P}}\left( { {\text{Preeclampsia}} _{ij} } \right)}}} \right) = \, \beta_{0j} \, + \,\beta_{1} *{\text{obesity}}_{ij} $$

$$ \, = \,\gamma_{00} \, + \,\beta_{{1}} *{\text{obesity}}_{ij} \, + \,{\text{u}}_{0j} $$

The key questions we would address are: (1) in addition to how the patient-level predictor affects the patient-level outcome, is there variability across different hospitals (which are associated with ‘clusters’ of patients), whether its effect on the patient-level outcome is *clinically important* (of a substantial magnitude that we think matters) and (2) whether the variability is *statistically significant* (that we have sufficient evidence that the relation is not due to chance alone)? To answer these, we need to use multi-level regression, or a comparable model, like GEE. If we determine that there are important differences across hospitals, and that they are influential on patient outcomes, then the following question is how influential the observed variability of certain practices or treatments across hospitals on patient outcomes is. This introduces a related, but distinct, concept of estimating the influence of ‘cluster-level variability’ on outcomes, which we can also assess using regression. In our example, we may be interested in (1) the fixed effect of obesity on preeclampsia, where cluster-variability is a secondary concern yet essential for accurate calculations of the standard errors of fixed effects or (2) the variability between clusters itself may be of primary interest, with fixed effects being a secondary consideration.

## Estimating the variability in a “clustered” outcome

Variability is a measure of dispersion around a number—how tight a distribution might be around a certain effect size; for example, the relative risk of preeclampsia when being cared for at one hospital or another. Variability can be observed in measurements within and across individuals (in this case, each individual acts as a cluster), and within and between clusters. Assessment of variability is important in health services research, allowing for understanding the associations between patient-level and cluster-level factors with individual health outcomes. We can use multi-level regression to estimate [5, 7–10]: (1) how much of the variability is attributed to the clusters’ characteristics; (2) how much of the variability is attributed to the differences between patients (i.e., within clusters); and (3) whether differences in outcomes among patients be explained by variability in clinical practice at cluster level. First, we will refine our aforementioned clinical question to a focused research question that seeks to quantify cluster-induced variability, and the variability-incorporated association of cluster-level variable with patient-level outcomes.

In the clinical scenario described above, provoked by the tragic death of both the mother and fetus, the research question might be: *what factors are associated with mortality among critically ill pregnant and post-partum women in acute care hospitals*. The association can have multi-factorial components at different levels; for example, in hierarchical order of bottom to top: the patient, the hospital, the health system (Fig. 2) [1]. We could even add another level, like the physician providing care, between the patient and the hospital, which could consequently reflect the reality of healthcare delivery. These factors suggest a hierarchical structure; yet researchers have often only considered variables at patient level. In our scenario, the primary outcome of interest is mortality, measured at patient level. Suppose the predictor of interest is the number of ICU beds, the type of ICU physician (e.g., an anesthetist, internist, or surgeon), or variability in ICU-admission practices. Variability in ICU-admission practices is expressed as the proportion of pregnant and post-partum women admitted to the ICU among all pregnant and post-partum women admitted to each hospital (i.e., as a hospital-level variable) [11]. There are reasons why a certain threshold for ICU admission may exist among providers: there may be hospital-specific ‘priority’ and advocacy for certain patient populations (e.g., trauma patients, or those recovering from surgery). Because we want to measure the influence of the predictor variables at the hospital level, other than the patient level, we must use multi-level regression to correctly estimate the effects corresponding to the association, and the potential causation between ICU admission *practice* and mortality of pregnant and postpartum women. Thus, a more nuanced research question might be: *what are the patient- and hospital-level factors associated with mortality among pregnant and postpartum women?*

## Estimating the influence of variability on outcomes using multi-level linear regression

When the outcome is continuous (e.g., blood pressure), there is an intuitive way to characterize variability in multi-level *linear* regression—the Intra-class Correlation Coefficient (ICC)—also referred as the Variance Partitioning Coefficient (VPC) [4, 5, 7–10, 12]. The ICC represents the degree to which individuals share a common experience due to “cluster” effects, i.e., the proportion of total variability from differences among (many) clusters. Equivalently, the ICC is the ratio of Between-Cluster Variability to Total Variability (variability from both between and within clusters):

$$\text{ICC} = \frac{\text{Between-Cluster Variability}}{\text{Between-Cluster Variability + Within-Cluster Variability}}$$

The ICC is, therefore, a measurement of the proportion of *Between*-Cluster Variability relative to total variability. In addition, “1 – ICC” represents the proportion of *Within*-Cluster Variability out of the total variability. When the ICC is minimal, the Between-Cluster differences are much smaller than the within cluster differences, demonstrating most of the variability is within the clusters (between patients). A large ICC means there is little variability within clusters and large variability between clusters. In such situation, we do not need multi-level regression, and instead can use simple regression, though it is good practice to still account for the hierarchical structure.

## Challenges in estimating between-cluster variability using multi-level logistic regression

The ICC is a way to understand the variability of individual outcomes in the multi-level context, but only when the outcome is continuous. If the outcome is binary (e.g., alive or dead), we employ multi-level *logistic* regression [8, 9]. In a multi-level logistic regression model, between-cluster variability and within-cluster variability are not directly comparable, as described below.

Unlike multi-level *linear* regression, in multi-level *logistic* regression the *scale* of variability at the lowest level (i.e., patient level) is different from that at higher levels (i.e., hospital level) [4, 5, 7–10, 12]. As explained previously, while the lowest-level variability is on the probability scale [e.g., mortality rate (p)], the higher-level variability is on the logistic scale because of the use of natural log on the odds of the outcome [e.g., log (odds of mortality) = log (p/1-p)]. Consequently, variability at both levels is not directly comparable due to different scales. Thus, it is not easy to fully “partition” variability at different levels in multi-level logistic regression; it is difficult to separate total variability into within-cluster and between-cluster variability.

To address this challenge, alternative statistical approaches have been developed to calculate ICC using multi-level logistic regression, including both *simulated* and *linear threshold* methods [9]. The simulated method converts the logistic scale of higher-level variability to the probability scale of the lowest-level variability. The linear threshold model method takes the reciprocal approach and converts the probability scale of the lowest-level variability to the logistic scale of higher-level variability. With a common scale for different levels of variability, the ICC can now be computed. In the latter case (the linear threshold model), the ICC is calculated with the following equation:

$$\text{ICC} = \frac{\text{Between-Cluster Variability}}{\text{Between-Cluster Variability} \, \text{+} \, {\pi }^{2}/3}$$

where π^2^/3 is the variance of the standard logistic distribution [8, 9]. There is inherent difficulty in interpreting variability at multiple levels with either the logistic- or probability-based approach given the complexity and non-linearity of these methods. The most common alternatives include *median odds ratio* and *interval odds ratio*.

## The median odds ratio and the interval odds ratio to estimate and interpret between-cluster variability in multi-level logistic regression

The median odds ratio (MOR) quantifies the variability between clusters (e.g., hospitals) using multi-level logistic regression [8, 9]. The main purpose of the MOR is to translate this variability between clusters into the odds ratio scale, which aids in easier interpretation of variability. The MOR’s definition is the median value of the distribution of the odds ratio between the cluster at higher risk and the cluster at lower risk selected randomly from two clusters (e.g., hospitals). For example, we randomly select two persons with same patient-level characteristics (say, present obesity) from two different hospitals and compute the odds ratio for the person with the higher risk of having the outcome versus the person with the lower risk. We then calculate the odds ratio from all pairs of persons to obtain a distribution of such odds ratios. The *median* OR is the median of this distribution. Alternatively, the MOR might be interpreted as the increased odds of an event when moving from a lower-risk *cluster* to a higher-risk cluster. The MOR is relatively easy to compute because it depends directly on the variability between clusters. The formula is the following [8, 9]:

$$\text{MOR= exp}\left(0.675\times \sqrt{2\times \text{Between Cluster-Variability}}\right)$$

where 0.675 is the 75th percentile of the standard normal distribution. Note that the MOR is always greater than or equal to 1, because the formula exponentiates a number greater than or equal to 0. An MOR of 1 indicates no between-cluster variability. It is insightful to compare the magnitude of the MOR, which quantifies the between-cluster variance, with the magnitude of the ORs for the fixed effects to understand the relative contribution of the random intercepts to the outcome’s overall variability, versus the fixed predictors.

To clinically understand what the MOR represents, we return to our example of determining the influence of patient-level, hospital-level (say, ‘variability in ICU admission practice’) and perhaps region-level variables on mortality [11]. An MOR of 2 would indicate that for two pregnant women with the same characteristics, the odds of dying may be twofold higher in one hospital compared to the other, given that we have considered other factors that may confound this association in our multivariable model. There are a number of examples of this concept in other medical and surgical specialties to illustrate the importance of using the MOR to estimate the influence of variability on outcomes [13–15]. For example, Wijeysundera and colleagues investigated predictors of pre-operative medical consultation for patients undergoing surgeries across 79 hospitals in Ontario. They concluded that patient-level factors are not meaningfully associated with pre-operative medical consult, yet there is variability of this practice among participated hospitals. Through multi-level logistic regression, they computed an MOR of 3.51, implying for two patients with very similar characteristics from different hospitals, one may be 3.51 times more likely to receive pre-operative medical consultation than another [15].

Both the ICC and MOR are tools to explore variability in multi-level regression. The ICC offers a more intuitive understanding of the variability in multi-level *linear* regression, while the MOR is more useful for multi-level *logistic* regression. While the ICC is chiefly a measure of between-cluster variability, it is influenced, by definition, by both between- and within-cluster variability, while the MOR reflects only between-cluster variability.

In frequentist statistics, the odds ratio is reported with a 95% confidence interval (CI), because this parameter is “fixed” and would lie within this interval, say, on average 95 times if the “experiment” was performed 100 times. The MOR, however, is random and has an associated distribution. Thus, the MOR should be reported with the Bayesian analogue of a 95% (CI), termed the 95% credible interval (Crl). When a parameter is “random” with a distribution around the MOR, the parameter lies in an interval of the distribution with a set probability. A 95% Crl of the MOR means that the MOR lies in this interval with 95% probability. Markov chain Monte Carlo methods are required to compute the MOR with a 95% Crl in multi-level logistic regression; however, further details are beyond the scope of this text [9].

The previous paragraph focused on MOR, we now consider the impact of ICU admission practice (a cluster-level variable) on outcomes by incorporating between-cluster variability. The Interval Odds Ratio (IOR) is a measure that estimates the range in which the true odds ratio of a variable at cluster-level lies, considering between-cluster variability. It is another type of interval used for the quantification of the impact of a variable at cluster level (e.g., hospital) on the outcome by incorporating between-cluster variability, in multi-level logistic regression. Note that the IOR does not describe the interval around a MOR, it reflects the size and variation of the OR of a cluster-level variable. An 80% IOR (IOR-80) is defined as the interval centered on the median of the distribution that comprises 80% of the odds ratio’s values. An 80% IOR is often recommended, because it covers a large portion of the odds ratios [8, 16]; however, the percentage is arbitrary, and other percentages could be considered.

Suppose that ICU admission practice (expressed as an annual incidence of ICU admissions, i.e., the number of ICU admissions divided by total number of hospitalized pregnant and postpartum women) is dichotomized as high versus low with the median value being the cutoff. When we compute a common OR of this ICU admission practice, an OR is calculated based on the comparison of mean odds in each group (high versus low). To compute the IOR of ICU admission practice, we might think about all possible pairs of subjects with the same covariates. For example, one woman is admitted to a hospital that more often admits pregnant and postpartum patients to the ICU (high group) and the other woman from a hospital that less frequently admits said patients to the ICU (low group). For each pair, we can calculate the OR between these subjects by accounting for the admission practice and variability between the two hospitals. We obtain the distribution of these ORs from considering all possible pairs. The IOR-80 is centered at the median of this distribution and covers 80% of the distribution of the ORs. Using this, we can calculate the lower and upper bounds of the IOR-80 with the following formulas that are similar to formulas we use for CIs:

$${\text{IOR}}_{lower}=exp\left(\beta -1.2816\times \sqrt{2\times \text{Between-Cluster Variability}}\right)$$

$${\text{IOR}}_{upper}=exp\left(\beta +1.2816\times \sqrt{2\times \text{Between-Cluster Variability}}\right)$$

In the above formulas, β is the regression coefficient for the cluster-level variable (e.g., ICU admission practice), Between-Cluster Variability is the cluster-level variance (variability), and − 1.2816 and 1.2816 represent the 10th and 90th percentiles of the standard normal distribution, respectively. Again, the IOR-80 is *not* a “confidence interval” around the MOR.

How do we interpret the IOR-80 in addition to a MOR? In our example, we explore the association of ICU admission practice for pregnant and postpartum women with mortality and measure variability induced by hospitals (cluster heterogeneity). We assess the MOR with the impact of the ICU admission practice among hospitals on mortality, in which simple odds ratios and 95% CIs and IOR-80 are used. We can employ multi-level logistic regression, to account for patient-level characteristics and the “clustering effect” of having many patients in a *certain number of unique hospitals*. In our case, we can interpret the different measures as follows:

- The Median Odds Ratio reflects the between-hospital variability or heterogeneity with regard to mortality; the 95% CrI reflects an interval where this Median Odds Ratio lies, with 95% probability.
- The Odds Ratio reflects the association of ICU admission practice with mortality; the 95% CI reflects the interval where this Odds Ratio lies when this experiment is repeated on multiple samples.
- The 80% Interval Odds Ratio (IOR-80) is a value centered at the median of the distribution of the ORs and covers 80% of the distribution, quantifying the association of ICU admission practice with mortality while accounting for “between cluster” (i.e., between hospital) variability.

How can we illustrate the importance of the above? Suppose both results show that women admitted to low ICU admission hospitals, compared with high ICU admission hospitals, have a higher odds of dying (say, OR = 2.34, with a 95% CI 1.77–3.09).

**Fig. 4:**
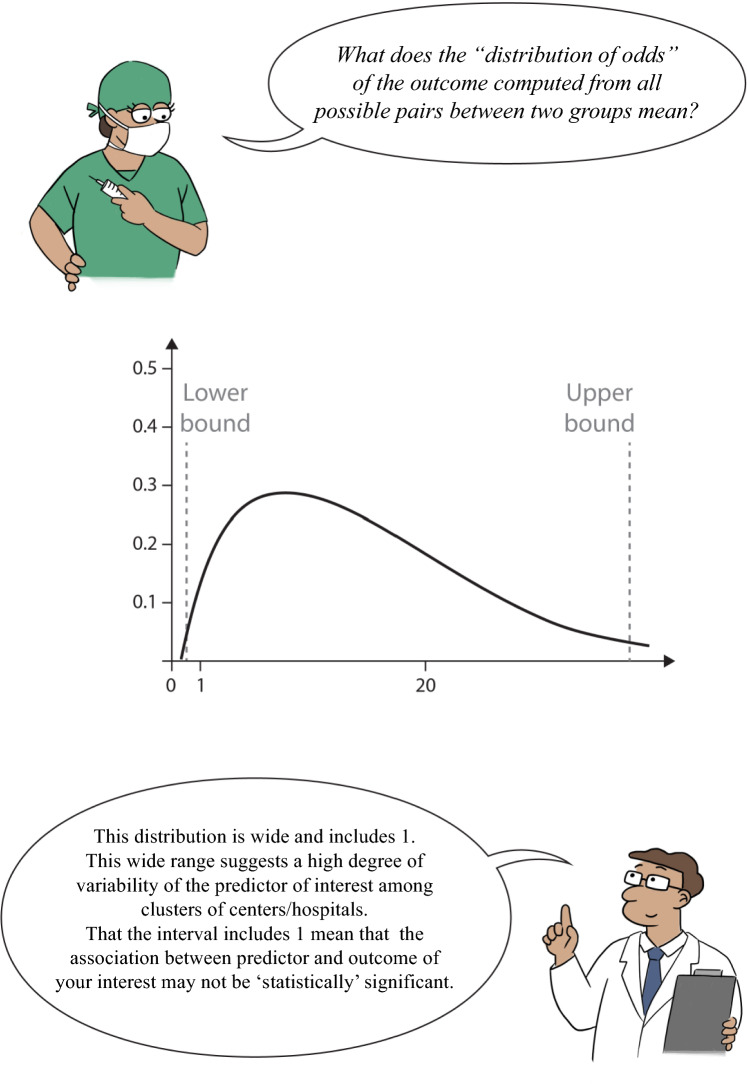
IOR-80 of Example 1

Assume we found the MOR to be 4.4 and the IOR-80 to be 0.14:39.4 (Fig. 4). This MOR is high, implying high variability among clusters. This is also reflected by the wide IOR. Since the interval includes 1, the IOR-80 informs ICU admission practices *may not* be significantly associated with mortality. This illustration implies substantial unexplained variability exists, which could be patient level or cluster level, or both.

**Fig. 5:**
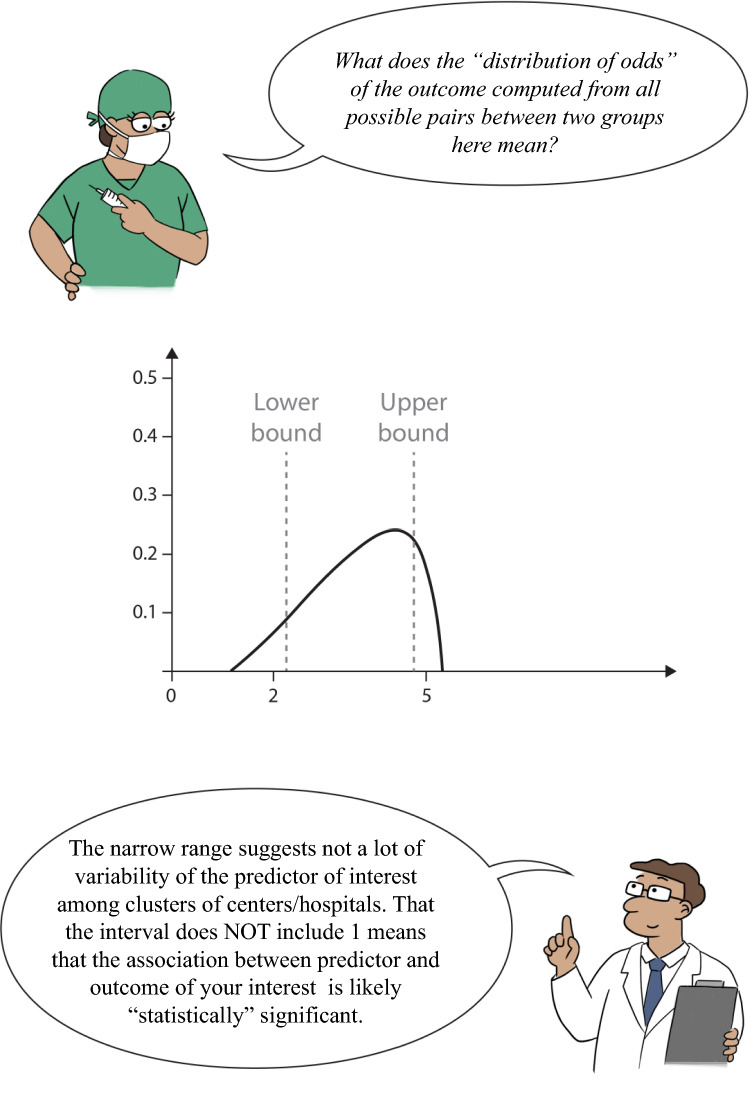
IOR-80 of Example 2

Suppose instead we found a MOR of 1.45 and IOR-80 1.164.73 (Fig. 5). This is a rather small odds ratio, implying little variability between clusters. The IOR-80 seems to be narrower due to little variability between clusters, just as the MOR implied. Moreover, the interval excludes 1, which means the influence of ICU admission practice is likely significantly associated with mortality.

## Discussion

In addition to being able to determine and interpret the influence of patient-level variables on a patient-level outcome, for continuous and binary outcomes, we have mechanisms to assess the influence of variables on different levels, organized in a hierarchy, on the outcome. Finally, we explored the concept of how variability among clusters might influence patient-level outcomes and investigated various methods to quantify and account for such influence. We have approached these topics with examples directly relevant to the field of anesthesia and with graphical visualizations to assist readers in accessing both fundamental and advanced concepts. We also wish to highlight an excellent, complementary hands-on tutorial that provides practical applications and instructional coding for foundational multi-level modeling [17].

In our example, we wished to investigate, among other factors, the association between ICU admission practice (the proportion of all hospitalized pregnant or postpartum women at each hospital who were admitted to the ICU) and mortality among these women. Given the data’s hierarchical structure, multi-level regression is the most appropriate methodology [11]. Through combining clinical experience and knowledge of pertinent literature, we included factors that might confound the relationship of interest, which is ICU admission practices. However, unless we consider, measure, and include all relevant factors, we could erroneously conclude that variability in ICU admission practices has a significant association with the outcomes, while it could be that, for example, patients from one hospital are systematically at higher risk from being older, having co-morbid conditions, having multiple gestations, etc. Unfortunately, in the real world, we often do not have data on many hospital-level or provider-level factors that could be influential. These, however, represent perhaps an opportunity for modification to the analysis, should they be found important.

For our own clinical question, we must address a few considerations related to practicality and confounding. First, how can we measure ICU admission practices for pregnant and postpartum women across many hospitals? Next, how can we estimate the association of variability in ICU admission practices between hospitals with clinical outcomes of patients? A hospital with a high-risk pregnancy birth center may tend to admit sicker pregnant and postpartum women than the average hospital, potentially leading to a higher incidence of ICU admissions and higher mortality in the hospital [18, 19]. In addition, the incidence of ICU admission might depend on the patient’s condition or severity. We might also assume that due to the higher incidence of ICU admission, the ICU physicians and co-workers may be knowledgeable about such patients and more skillful at treating them, potentially reducing morality. To incorporate the rate of ICU admissions as a proxy of ICU admission practices, we should incorporate a risk adjustment tool so that a patient’s background medical condition (comorbidities, for example) and current illness severity can be accounted for—i.e., their influence adjusted by including them as patient-level variables in our multi-level model. A maternal comorbidity index has recently been described as a risk adjustment tool for hospitalized pregnant and postpartum women, and has been validated in second external dataset [20–22]. The use of a maternal comorbidity index in the multi-level regression may assist in isolating the potential influence of ICU admission practices on the outcome of interest among pregnant and postpartum women [11, 23].

Because our primary outcome is mortality (a binary variable), we have to employ a multi-level *logistic* regression. We can compute the MOR, which can help estimate between-cluster (hospital) variability. Anecdotally and from our research, we know hospitals often have different ICU admission rates for pregnant and postpartum women. Therefore, we should understand how to estimate the effect size of this variability on the outcome (mortality). However, the MOR does not consider within-cluster (patient-level) variability. In this case, we could utilize multi-level models with an appropriate hierarchical structure to capture and account for within-cluster variability. We could also categorize hospitals into several groups according to their ICU admission rates and compute the MOR of each group. Comparing the MORs across groups could inform whether between-cluster heterogeneity in mortality may change according to different ICU admission practices. We could also use IOR-80 to measure the association between ICU admission practices and mortality, accounting for the “between cluster” (i.e., between hospital) variability. These approaches allow us to see how differences in ICU admission practices might explain variability in mortality [11].

## Conclusion

It is important for clinicians and researchers to understand factors that are associated with and may influence patient outcomes. This enables us to better identify patients who are at risk for bad outcomes, which in turn may influence our patient care. This could also help us identify modifiable factors—for patients, for hospitals, for healthcare providers—that can be addressed to improve patient care and outcomes. Estimating the association of potential predictor variables with the outcome could be challenging as data and analyses are prone to different types of bias and confounding. We reviewed regression as a common method derive the independent influence of variables and provided examples when multi-level regression is required. Finally, we presented methods to measure variability of care delivery between hospitals or providers or other clusters and estimate the influence of such variability on patient outcomes. By deepening our understanding of these techniques, we can enhance our ability to identify and target modifiable factors for improving patient care and outcomes.
